# Effectiveness of Computer-Based Psychoeducational Self-Help Platforms for Eating Disorders (With or Without an Associated App): Protocol for a Systematic Review

**DOI:** 10.2196/60165

**Published:** 2024-11-04

**Authors:** Alessandra Gentile, Yosua Yan Kristian, Erica Cini

**Affiliations:** 1 Department of Child & Adolescent Psychiatry Institute of Psychiatry, Psychology & Neuroscience King’s College London London United Kingdom; 2 Division of Medicine University College London London United Kingdom; 3 East London NHS Foundation Trust London United Kingdom

**Keywords:** self-help, online self-help, eating disorders, anorexia nervosa, psychoeducational intervention, psychoeducation, binge eating, anorexia, bulimia, access to care, patient education, patient self-help

## Abstract

**Background:**

Access to psychological health care is extremely difficult, especially for individuals with severely stigmatized disorders such as eating disorders (EDs). There has been an increase in children, adolescents, and adults with ED symptoms and ED, especially following the COVID-19 pandemic. Computer-based self-help platforms (± associated apps) allow people to bridge the treatment gap and receive support when in-person treatment is unavailable or not preferred.

**Objective:**

The aim of this systematic review is to evaluate the effectiveness of computer-based self-help platforms for EDs, some of which may have associated apps.

**Methods:**

The proposed systematic review will follow the PRISMA (Preferred Reporting Items for Systematic Reviews and Meta-Analyses) guidelines. This review will report and evaluate the literature concerning the efficacy of self-help platforms for EDs. Articles were obtained from the Ovid MEDLINE, Embase, Global Health, and APA PsycInfo. The inclusion criteria included research with original data and gray literature; research evaluating the efficacy of web-based psychoeducational self-help platforms for EDs; people with an ED diagnosis, ED symptoms, at risk of developing EDs, or from the general population without ED-related behaviors; pre– and post–computer-based ± associated apps intervention clinical outcome of ED symptoms; pre– and post–computer-based ± associated apps intervention associated mental health difficulties; and literature in English. The exclusion criteria were solely guided self-help platforms, only in-person interventions with no computer-based ± associated apps comparison group, only in-person–delivered CBT, self-help platforms for conditions other than eating disorders, systematic reviews, meta-analyses, posters, leaflets, books, reviews, and research that only reported physical outcomes. Two independent authors used the search terms to conduct the initial search. The collated articles then were screened by their titles and abstracts, and finally, full-text screenings were conducted. The Cochrane Risk of Bias 2 tool will be used to assess the risks of bias in the included studies. Data extraction will be conducted, included studies will undergo narrative synthesis, and results will be presented in tables. The systematic review will be submitted to a peer-reviewed journal.

**Results:**

The authors conducted a database search for articles published by May 31, 2024. In total, 14 studies were included in the systematic review. Data charting, synthesis, and analysis were completed in Microsoft Excel by the end of July 2024. Results will be grouped based on the intervention stages. The results are expected to be published by the end of 2024. Overall, the systematic review found that computer-based self-help platforms are effective in reducing global ED psychopathology and ED-related behaviors.

**Conclusions:**

Self-help platforms are helpful first-stage resource in a tiered health care system.

**Trial Registration:**

PROSPERO CRD42024520866; https://tinyurl.com/5ys2unsw

**International Registered Report Identifier (IRRID):**

DERR1-10.2196/60165

## Introduction

### Background

Eating disorders (EDs) are a group of heterogeneous psychiatric disorders that are characterized by irregular eating behaviors [1]. Research demonstrated that between 5.5% and 17.9% of women and 0.6% and 2.4% of men meet the *DSM-5* (*Diagnosis and Statistical Manual of Mental Disorders* [Fifth Edition]) criteria for EDs [2]. Furthermore, gender and sexual minorities are demonstrated to be particularly at risk for developing EDs, with anorexia nervosa and bulimia being the most prevalent [2-4]. Compared with any other psychiatric conditions, anorexia nervosa has the highest suicidality and mortality rates and lowest quality of life levels, highlighting the importance of urgency of care [5,6]. Despite this, merely one-fourth of individuals with ED symptoms or developed EDs access care [7]. EDs have been demonstrated to negatively impact the psychological, cognitive, physical, and social development of individuals, which evidences the need for accessible resources, such as self-help platforms, which support individuals and prevent, treat, and avoid the relapse of EDs [8].

During the COVID-19 pandemic, there was an increase in children and adolescents struggling with ED symptoms and EDs, leading to a surge in waiting times [9,10]. Whilst face-to-face treatments such as ED-focused family therapy, CBT-E have been demonstrated to effectively reduce ED symptoms for eating disorders, many previously in-person psychoeducational support systems transitioned to computer-based self-help platforms [11,12]. Computer-based self-help platforms ± associated apps are stand-alone support websites or mobile apps which do not require the presence or assistance of trained professionals [8]. Self-help platforms overcome issues related to the help-seeking stigma, geographical isolation, and time constraints, which have been demonstrated as factors which prevent help-seeking [13], though they rely on the individual’s motivation to access support. Health care systems have evolved to provide a resource-efficient framework for communities to provide stepped support for individuals depending on their level of need. One such framework is the THRIVE framework [14].

THRIVE provides service providers with a set of principles to help guide their care approaches by distinguishing between 5 stages of care provision: “thriving,” “getting advice,” “getting help,” “getting more help,” and “getting risk support.” Self-help platforms are the initial stage of accessing support, and when more support is needed, then the initial stage of “getting advice” to “getting help” [14]. Self-help platforms are available for the general public and provide psychoeducational information for those requiring additional resources or support [14]. Although self-help platforms are good prevention strategies, research has demonstrated that individuals are significantly more satisfied when self-help platforms are provided in combination with in-person interventions [15].

Psychoeducational self-help computer-based platforms ± associated apps aid the understanding of ED’s causes, risk factors, and potential consequences for individuals and their families with the aim of developing effective coping mechanisms [16,17], but they need to be able to recognize the problem and be motivated to seek help. Self-help platforms allow for early recognition and early skills development to people who identify with at risk or disordered eating symptoms. [18]. A variation of approaches can be used in addition to the psychoeducation, such as implementing cognitive behavioral therapy (CBT) techniques, dissonance-based interventions (DBI), and motivation enhancement therapeutic techniques (MET). The specific aims and target groups for the therapeutic techniques can be found in Table 1.

**Table 1:** Cognitive behavioral therapy, dissonance-based intervention, and motivation enhancement therapy.

Therapeutic design	Aim	Target group
CBT^a^	Focuses on the relationship between thoughts, behaviors, and feelings. For ED^b^, it addresses one’s thoughts and behaviors related to their irregular eating patterns [19].	Effective for all age groups, targets a variety of disorders, including ED.
DBI^c^	Challenges society and media-propagated body ideals [20]. It is often most effective when implemented during key developmental stages.	Effective for all age groups, most effective for individuals who have maladaptive and conflicting attitudes and behaviors, often used for EDs and substance abuse issues.
MET^d^	Urges people with ED’s to make changes to their behaviors and cognitive processes [21].	Effective for all age groups, MET often combined with CBT to enhance individuals’ motivation to change maladaptive behaviors and relationship with food.

^a^CBT: cognitive behavioral therapy.

^b^ED: eating disorder.

^c^DBI: dissonance-based intervention.

^d^MET: motivation enhancement therapy.

Previous systematic reviews have demonstrated guided computer-based self-help platforms ± associated apps as effective in reducing ED core symptoms and ED-related behaviors by altering maladaptive behaviors and changing the individual perseverative thinking, thin idealization, body dissatisfaction, depression, quality of life, and lack of motivation to change [17,22]. However, to the knowledge of the authors, there has not yet been a systematic review evaluating the effectiveness of solely self-help platforms for EDs [17,23,24]. Therefore, the current systematic review aims to evaluate the literature on the effectiveness of ED self-help platforms. The effectiveness of self-help platforms will be primarily measured by the platform’s ability to reduce ED symptomology. Second, the effectiveness will be evaluated based on the platform’s ability to decrease ED-related behaviors such as perseverative thinking, thin idealization, body dissatisfaction, depression, quality of life, and resistance to change.

### Objective

This systematic review aims to explore literature which has evaluated the following research questions:

1. Are computer-based psychoeducational self-help platforms ± associated apps effective in preventing the onset of EDs?
2. Are computer-based psychoeducational self-help platforms ± associated apps effective in reducing the core ED symptoms?
3. Are computer-based psychoeducational self-help platforms ± associated apps effective in improving the long-term effects of EDs?

This systematic review will follow the PRISMA (Preferred Reporting Items for Systematic Reviews and Meta-Analyses) guidelines to evaluate the literature regarding the effectiveness of web-based psychoeducational and self-help platforms for EDs.

## Methods

### Overview

A systematic review was conducted to explore the literature regarding the effectiveness of computer-based psychoeducational self-help platforms ± associated apps for people with ED symptoms and developed EDs. The reporting of this systematic review abided by the PRISMA guidelines [25].

### Eligibility Criteria

#### Population

This systematic review did not have a set age restriction for the population. The systematic review considered research that had screened participants at baseline level, before the intervention, and following intervention. The population included individuals with no ED behavioral patterns, individuals who were at-risk of developing ED’s, and individuals with any diagnosed ED.

#### Intervention

This review included literature examining the clinical effectiveness of computer-based psychoeducational self-help platforms ± associated apps. The comparison groups were treatment as usual.

#### Outcome

Clinical effectiveness was defined as a decrease in ED symptoms, ED related behaviors, and increase in weight for those with a restrictive eating disorder.

#### Context

This review considered studies conducted in any geographical context using any computer-based intervention platforms ± associated apps for EDs.

#### Types of Evidence Source

This review considered studies with all types of research designs (eg, randomized controlled trials, quasi-experimental and case-control studies, clinically on-controlled studies, and observational studies) and studies using qualitative, quantitative, or mixed methods. Gray literature was analyzed and included as appropriate. Furthermore, only studies published in English was included.

The inclusion and exclusion criteria are mentioned in Textbox 1.

**Inclusion and exclusion criteria.**

**Inclusion criteria**

- Research with original data, including gray literature.
- Research evaluating the efficacy of web-based psychoeducational self-help platforms for eating disorders (EDs).
- People with ED diagnosis.
- People with ED symptoms.
- People at risk of developing EDs.
- People from the general population without ED-related behaviors.
- Research which examined the pre- and post-computer-based intervention ± associated apps clinical outcome of ED symptoms.
- Research which examined the pre– and post–computer-based intervention ± associated apps associated mental health difficulties.
- Literature in English.

**Exclusion criteria**

- Guided self-help platforms.
- Only in-person interventions with no computer-based ± associated apps comparison group.
- Only in-person delivered cognitive behavioral therapy.
- Self-help platforms for conditions other than Eds.
- Systematic reviews.
- Meta-analyses.
- Posters.
- Leaflets.
- Books.
- Reviews.
- Research that only reported physical outcomes.

### Search Strategy

A systematic search was conducted using Ovid MEDLINE, Embase, Global Health, and APA PsycInfo up to the established date of May 31, 2024. A manual search of references was conducted using Google Scholar to identify alternative literature. Furthermore, a reference list search will be conducted to identify any other potential literature that can be included.

Author AG conducted an initial search using Ovid on May 31, 2024, using preliminary search terms developed by AG and YYK and reviewed by EC. AG used the search terms “Eating Disorder* OR Anorexia OR Anorexia Nervosa OR Bulimia OR Binge eating OR Avoidant restrictive food intake disorder OR ARFID OR Otherwise specified feeding or eating disorder OR OSFED” and “Intervent* OR Treatment* OR Psychoedu*” and “ICBT OR Internet cognitive behavioral therapy OR self-help OR digital OR online.” The search terms were identified based on previous literature and key relevant terms relating to ED self-help platforms. The table depicting the search terms and the results can be seen in the Multimedia Appendix 1.

### Study Selection

After the completion of the search, studies were exported to EndNote and duplicates were removed. Articles were then initially screened independently by AG and YYK by titles and abstracts. Following this, the authors full text screened the literature and collected research which abided by the inclusion and exclusion criteria. The reviewers met to discuss the pertinent articles. EC was contacted to provide further insight and resolve any disagreements when AG and YYK could not reach an agreement following the discussion. Literature that did not meet the inclusion criteria was excluded from the systematic review.

### Data Extraction

Data extraction will be completed by the researchers capturing key aspects of studies that fit the inclusion criteria. The key aspects will be initially recorded on a Microsoft Excel spreadsheet. The aspects extracted will include primary author name and published date, sample population demographics, follow-up times and sample size, outcome measure, intervention, key findings, and overall risk of bias. A draft of the table is presented in Textbox 2. However, this draft may be modified in the final review. The data will be inputted by AG and YYK independently for each included study, to assess the studies eligibility. In cases of any disagreements, the reviewers will discuss to reach a consensus. EC will be consulted in the case of uncertainty or unsolved disagreement.

**Table components draft.**

**Components**

- Authors, date.
- Baseline sample (N), female %, and mean age.
- Follow-up times and sample size (% of initial sample).
- Outcome measure.
- Intervention.
- Key findings.
- Risk of bias.

### Data Synthesis and Quality Assessment

The findings of the systematic review will be initially summarized in a Microsoft Excel table and then narratively synthesized and analyzed based on the guidelines specified by Popay et al [26]. Included studies used diverse methods to analyze primary and secondary outcomes (ie, clinical effectiveness and associated mental health difficulties). Therefore, the reliability of the questionnaires will be analyzed and reported. Quality assessment will be performed using the Grading of Recommendations Assessment, Development and Evaluation approach.

### Risk of Bias

Following the initial search, the primary author found all included literature was randomized controlled trials; therefore, the Cochrane Risk of Bias 2 tool was selected. If additional research methods are detected in the included literature, the risk of bias tool may be revised. The Cochrane Risk of Bias 2 tool will be used to ensure that all questionnaires are approximately similar in validity and reliability. Detailed information regarding the selection procedure will be presented in a PRISMA flow diagram. Where applicable, common findings amongst the research will be identified and reported.

The primary outcomes will be reported in terms of the web-based self-help platforms’ effectiveness in decreasing the clinical symptoms of eating disorders in participants. Secondary outcomes will be analyzed in terms of associated mental health difficulties (ie, perseverative thinking, body dissatisfaction, thin idealization, fear of becoming fat, preoccupation of food and weight, motivation to change their weight, self-esteem, depression, and quality of life).

AG and YYK will independently analyze potential biases of the studies using the Cochrane Risk of Bias tool. The risk of bias tool assessment examined 5 domains of potential risk of bias namely, randomization, divergence from the intended intervention, analysis of missing data, measurement of outcomes, and selection of reported results.

### Missing Values

Self-help platforms have been demonstrated to have high dropout and attrition rates. Therefore, the bias caused by missing values will be determined at the discretion of the authors. The authors will reflect the impact of the missing values on the Cochrane Risk of bias 2. Meta-analysis will not be performed due to high heterogeneity of the outcomes of studies.

### Ethical Considerations

No patients or members of the public were involved in the development of this protocol or will be involved in the final scoping review. Nevertheless, including qualitative studies should give a valuable insight into patient and carer perceptions and experiences with intensive community and home treatments. Ethical approval will not be required for this review as the data will be obtained from publicly available sources.

## Results

**Figure 1:**
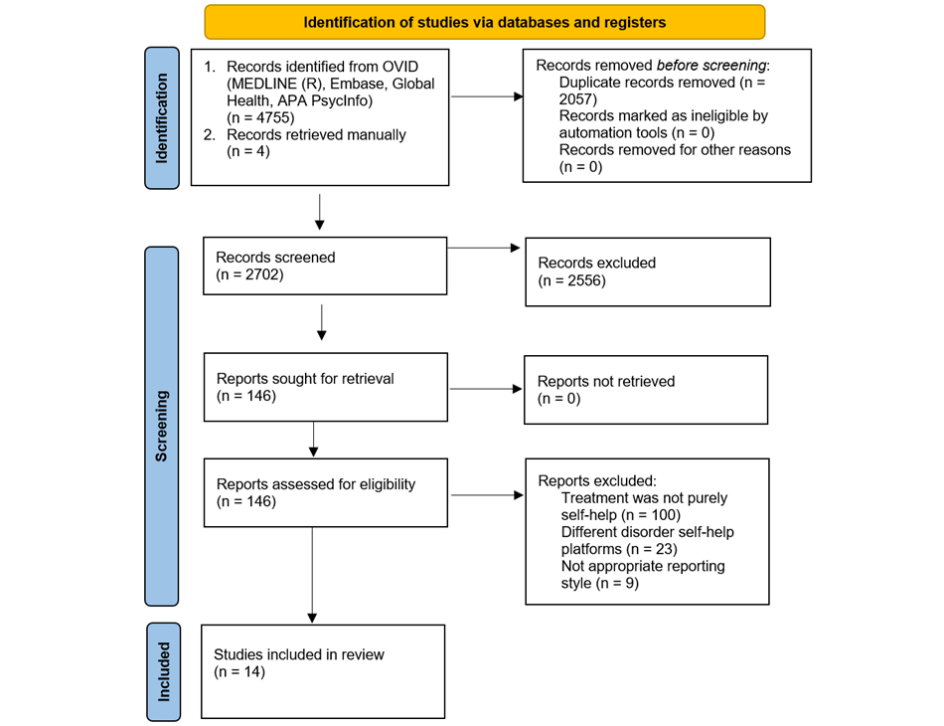
The PRISMA (Preferred Reporting Items for Systematic Reviews and Meta-Analyses) flowchart.

This systematic review explores the effectiveness of web-based self-help interventions for the prevention and management of EDs. Data collection was conducted until May 31, 2024. Overall, 14 studies were collated, which were in line with the inclusion and exclusion criteria of this systematic review. Due to varying population groups, the data will be divided based on the stepped intervention approach taken. Studies conducted with the general population participants will be grouped under primary prevention, studies conducted with participants who are at risk of developing EDs will be grouped under secondary prevention, and studies conducted with participants who have clinically significant ED symptoms will be grouped under tertiary prevention. Data charting is provided in Textbox 2 above. The studies will be narratively synthesized, and the results will be presented in the form of a systematic review report. Data analysis and write-up were completed by the end of July 2024, and the result is expected to be published by the end of 2024. The PRISMA flow chart can be seen in Figure 1.

## Discussion

This is the first systematic review solely exploring existing literature the effectiveness of unguided computer-based psychoeducational self-help platforms ± associated. Whilst previous systematic reviews compared guided with nonguided computer-based psychoeducational self-help platforms ± associated apps, this systematic review evaluates the effectiveness of self-help platforms across the age range.

One of the biggest challenges in conducting unguided computer-based psychoeducational self-help platforms ± associated apps is the high dropout rate. This systematic review discusses the issue and mentions the effectiveness of the treatment in different ethnic population. The effectiveness of different unguided self-help platforms to ED psychopathologies and ED-related behaviors will be analyzed.

There are some strengths related to this systematic review. This systematic review will provide an overview of the effectiveness of the computer-based psychoeducational self-help platforms ± associated apps and the feasibility of implementing the platforms in clinical settings. It is expected that this systematic review will provide directions for future research and tactics to improve already existing web-based self-help platforms. Furthermore, the findings of this review will be reported according to the rigorous guidelines for systematic reviews outlined in the PRISMA. In addition, 2 independent researchers conducted searches and data extraction, increasing the findings’ internal validity. As a limitation to the systematic review, there is a high dropout rate found from the studies included. Consequentially, the systematic review will have a limited overview of the validity of the efficacy of computer-based psychoeducational self-help platforms ± associated apps. Furthermore, a meta-analysis might not be performed due to the high risk of heterogeneity in the studies. The review’s findings will be disseminated through conference presentations and peer-reviewed publications.

## Appendix

Multimedia Appendix 1 Search results from OVID databases (MEDLINE, Embase, Global Health, and APA PsycInfo).

## Abbreviations

CBT: cognitive behavioral therapy
DBI: dissonance-based intervention
*DSM-5*: *Diagnostic and Statistical Manual of Mental Disorders (Fifth Edition)*
ED: eating disorder
MET: motivation enhancement therapy
PRISMA: Preferred Reporting Items for Systematic Reviews and Meta-Analyses

## Data Availability

The datasets used after this systematic review results are completed will be available from the corresponding author upon reasonable request.
