# Supplementary material for: Effectiveness of Computer-Based Psychoeducational Self-Help Platforms for Eating Disorders (With or Without an Associated App): Protocol for a Systematic Review
Source: JMIR Res Protoc. 2024 Nov 4;13:e60165. doi: 10.2196/60165 (PMC11574503; doi:10.2196/60165)
Supplement: Multimedia Appendix 1 [file resprot_v13i1e60165_app1.pdf]

Search terms from OVID for MEDLINE (R), Embase, Global Health, and APA PsychInfo from inception to 31 May 2024

#	Search terms	Results
1	intervent*.mp. [mp-ti, ab, hw, tn, ot, dm, mf, dv, kf, fx, dq, bt, nm, ox, px, rx, an, ui, sy, ux, mx, cw, tc, id, tm]	4228073
2	treatment*.mp. [mp=ti, ab, hw, tn, ot, dm, mf, dv, kf, fx, dq, bt, nm, ox, px, rx, an, ui, sy, ux, mx, cw, tc, id, tm]	16822404
3	psychoedu*.mp. [mp=ti, ab, hw, tn, ot, dm, mf, dv, kf, fx, dq, bt, nm, ox, px, rx, an, ui, sy, ux, mx, cw, tc, id, tm]	38793
4	ICBT.mp. [mp=ti, ab, hw, tn, ot, dm, mf, dv, kf, fx, dq, bt, nm, ox, px, rx, an, ui, sy, ux, mx, cw, tc, id, tm]	3269
5	internet cognitive behavioral therapy.mp. [mp=ti, ab, hw, tn, ot, dm, mf, dv, kf, fx, dq, bt, nm, ox, px, rx, an, ui, sy, ux, mx, cw, tc, id, tm]	114
6	self-help.mp. [mp=ti, ab, hw, tn, ot, dm, mf, dv, kf, fx, dq, bt, nm, ox, px, rx, an, ui, sy, ux, mx, cw, tc, id, tm]	61062
7	digital.mp. [mp=ti, ab, hw, tn, ot, dm, mf, dv, kf, fx, dq, bt, nm, ox, px, rx, an, ui, sy, ux, mx, cw, tc, id, tm]	546581
8	online.mp. [mp-ti, ab, hw, tn, ot, dm, mf, dv, kf, fx, dq, bt, nm, ox, px, rx, an, ui, sy, ux, mx, cw, tc, id, tm]	732960
9	eating-disorder*.mp. [mp=ti, ab, hw, tn, ot, dm, mf, dv, kf, fx, dq, bt, nm, ox, px, rx, an, ui, sy, ux, mx, cw, tc, id, tm]	135218
10	anorexia.mp. [mp=ti, ab, hw, tn, ot, dm, mf, dv, kf, fx, dq, bt, nm, ox, px, rx, an, ui, sy, ux, mx, cw, tc, id, tm]	179602
11	anorexia nervosa.mp. [mp=ti, ab, hw, tn, ot, dm, mf, dv, kf, fx, dq, bt, nm, ox, px, rx, an, ui, sy, ux, mx, cw, tc, id, tm]	67317
12	bulimia.mp. [mp-ti, ab, hw, tn, ot, dm, mf, dv, kf, fx, dq, bt, nm, ox, px, rx, an, ui, sy, ux, mx, cw, tc, id, tm]	49331
13	binge eating.mp. [mp=ti, ab, hw, tn, ot, dm, mf, dv, kf, fx, dq, bt, nm, ox, px, rx, an, ui, sy, ux, mx, cw, tc, id, tm]	34462
14	1 or 2 or 3 or 4 or 5	19536361
15	6 or 7 or 8	1302740
16	9 or 10 or 11 or 12 or 13	286650
17	14 and 15 and 16	4755
18	remove duplicates from 17	2698
